# Optimizing generalized anxiety disorder screening in young adults perinatally affected by HIV: A psychometric analysis

**DOI:** 10.1016/j.xjmad.2024.100066

**Published:** 2024-03-30

**Authors:** Corey Morrison, Claude A. Mellins, Clayton Synder, Eileen Shea, Luke Kluisza, Reuben Robbins, Ohemaa Poku, Prudence Fisher, Elaine Abrams, Andrew Wiznia, Laura Mufson

**Affiliations:** a HIV Center for Clinical and Behavioral Studies, New York State Psychiatric Institute and Columbia University, 1051 Riverside Dr #15, New York, NY 10032, USA; b Department of Psychiatry, Mental Health Data Science, Columbia University Medical Center, 1051 Riverside Dr, New York, NY 10032, USA; c Child and Adolescent Psychiatry, New York State Psychiatric Institute and Columbia University, 1051 Riverside Drive, New York, NY 10032, USA; d Mailman School of Public Health, Columbia University, 722 W 168th St, New York, NY 10032, USA; e Jacobi Medical Center, Albert Einstein College of Medicine, New York, NY, USA

**Keywords:** HIV, Anxiety, GAD-7, Validation, Young adults

## Abstract

**Background:**

Generalized Anxiety Disorder (GAD) is prevalent among people with HIV and is associated with adverse health outcomes. This study investigates the suitability of the Generalized Anxiety Disorder Scale-7 item (GAD-7) screening tool and its 2-item (GAD-2) version for use in young adults with perinatally-acquired HIV (YAPHIV) and young adults perinatally exposed to HIV but uninfected (YAPHEU).

**Methods:**

Data come from the 7th follow-up interview (FU7) from a longitudinal study of youth with PHIV and PHEU, first recruited when 9–16 years. The GAD-7 was administered along with a diagnostic psychiatric interview (DISC-IV). Receiver Operating Characteristic analysis assessed accuracy, sensitivity, and specificity of the GAD7 and GAD-2. Subgroup analyses considered HIV status, ethnicity, and race.

**Results:**

At FU7, participants (n = 204) were ages 20–29; 54% female; and the majority African-American and/or Latinx. 12% met diagnostic criteria for GAD. Recommended GAD-7 (>10) and GAD-2 (>3) cut-scores showed suboptimal sensitivity (0.52 and 0.48, respectively) and high specificity (0.91 and 0.90, respectively). Lowering cut-scores (GAD-7 >6 and GAD-2 >2) improved sensitivity (0.76 and 0.80) while sacrificing specificity (0.77 and 0.78). Stratified analyses by HIV status revealed similar accuracy in YAPHIV and YAPHEU. Race/ethnicity did not significantly affect cut-scores.

**Discussion:**

Anxiety disorders are common in YAPHIV, and efficient screening is essential. While the GAD-7 and GAD-2 show promise, recommended cut-scores may not be optimal. Lowering cut-scores may enhance sensitivity without losing clinical utility. Further research is needed to refine cut-scores based on demographic characteristics and in global contexts, ensuring effective anxiety screening in this population.

## 1. Background

Generalized Anxiety Disorder (GAD) is a common mental health disorder in people with HIV, which has significant implications for their overall well-being and treatment outcomes [1–3]. GAD, according to Diagnostic and Statistical Manual of Mental Disorders (DSM) criteria, is characterized by excessive, persistent, and difficult to control fear or worry [4]. 12-month GAD prevalence in people with HIV has been estimated to range between 6.5–20% [5], which is higher than that found for the general population (5.7%) [6]. Among the general population, GAD has been found to significantly impair health-related quality of life [7,8], and negatively influence help-seeking behavior, self-care, and interpersonal functioning [9,10]. In people with HIV, not only does GAD significantly lower quality of life [11–13], it is also associated with suboptimal antiretroviral (ART) adherence, lower sustained viral suppression [14] and HIV care retention [1], as well as engagement in sexual risk behavior [15], thereby increasing the risk for HIV-related morbidity and HIV transmission. Thus, identifying and effectively treating mental health disorders, such as GAD, in people with HIV is recognized as a necessary step to ending the HIV epidemic [3].

Young people with HIV are a critical population needing evidence-based treatments for comorbid HIV and mental disorders, including those with perinatally-acquired HIV [3,16,17]. In the United States, the vast majority of the approximately 12,000 people with early-acquired HIV (the vast majority with perinatally-acquired HIV), are entering late adolescence and young adulthood [18,19], a vulnerable developmental period frequently accompanied by the onset of GAD [6,20]. Furthermore, most young adults with perinatally-acquired HIV (YAPHIV) are Black and Latinx and come from communities affected by poverty, neighborhood violence, substance use, and health care disparities, all of which can affect health and behavioral outcomes [21–23]. Thus, YAPHIV may be at a higher risk of experiencing anxiety and other negative behavioral health outcomes [24,25]. Unfortunately, screening and treatment for GAD in these young people as well as in other age groups living with HIV has received little attention [26–28]. Young adults perinatally exposed to HIV but uninfected (YAPHEU) share many sociodemographic characteristics with YAPHIV [29]. Prior literature has shown YAPHEU to have elevated rates of mental health disorders including GAD [30,31] while being less likely to receive mental healthcare than YAPHIV [25].

The Generalized Anxiety Disorder Scale-7 (GAD-7) [28], is a brief, widely used screening tool for GAD, which was developed as a response to the recognized paucity of brief, clinically appropriate screening instruments for anxiety as compared to measures for other common mental disorders, particularly depression [32]. The GAD-7 comprises seven items reflecting DSM-defined symptoms for GAD [4]. The GAD-2, an abbreviated version, consists of the first two items from the GAD-7. The GAD-7 and GAD-2 have both been widely used in both primary care and population-based studies [33,34]. Previous validation studies have shown that the recommended cut-score of 10 or higher on the GAD-7 has a sensitivity of 89% and a specificity of 82% for detecting GAD [28] in adults, as evaluated by the Structured Clinical Interview for DSM-IV (SCID;[35]). Similarly, the recommended cut-score of 3 or higher on the GAD-2 was found to have a sensitivity of 86% and a specificity of 83% [33]. While the performance of the GAD-7 as a screener for adolescents and young adults has been examined by many investigators [28,34,36–38] with robust support for its use, most of the samples used were Caucasian, and less is known about the psychometric properties of the screener in other racial or ethnic groups or in populations known to have high rates of mental health disorders, such as young adults living with HIV. One study analyzing differential item functioning in the GAD-7 found that Black participants who reported a high latent GAD trait tended to score lower on specific GAD-7 items 1, 5, and 6, than white and Latinx participants reporting similar latent levels of GAD [39], which could lead to under-identification of GAD among Black populations using the GAD-7 due to underreporting of severity on these items. It remains unclear whether the recommended cut-point of 10 is optimal for the detection of GAD in vulnerable HIV-affected populations, a majority of whom are from Black and Latinx backgrounds in the United States [21].

While the GAD-7 and GAD-2 have been used in the United States and in low- and middle-income countries [1,40–42], the accuracy of the recommended GAD-7 and GAD-2 cut-scores in people with HIV, including YAPHIV, is unclear. A prior study on the use of the Patient Health Questionnaire-9 (PHQ-9), a similarly constructed depression screening instrument, found that recommended cut-scores were not optimal for YAPHIV [43]. However, to our knowledge, there are no comparable studies looking at the GAD-7 and GAD-2. This research gap is notable given high rates of anxiety disorders in YAPHIV and YAPHEU, who are exposed to other stressors, and are primarily racial and ethnic minorities [30].

To address this knowledge gap, we investigated the effectiveness of the recommended GAD-7 and GAD-2 cut-scores, 10 and 3, respectively, in detecting DSM-defined GAD in a sample of YAPHIV and a comparison group of YAPHEU who otherwise share sociodemographic characteristics with YAPHIV (race, ethnicity, birth mother with HIV) [16,30,44]. The sample is part of a longitudinal cohort study on the psychiatric and social impact of perinatally-acquired HIV infection.

## 2. Methods

The Child and Adolescent Self-Awareness and Health study (CASAH) is an ongoing longitudinal study of youth with perinatally-acquired HIV and perinatal HIV-exposure. Study enrollment occurred between 2003–2008, with 340 participants between the ages of 9–16 years recruited from four medical centers in New York City. The primary objectives of the study are to examine psychiatric and psychosocial outcomes in this population and their relationship with HIV, young adult transition milestones, and other psychological, biomedical, neurocognitive, and social determinants of health and behavioral health. Detailed information on study procedures has been previously reported [30,44,45]. Briefly, the study identified 443 eligible participants at four medical clinics, of which 340 (77%) were successfully enrolled. Inclusion criteria were: perinatal HIV exposure, cognitive ability to understand procedures and provide assent to complete the interview, youth living with caregiver with legal capacity to sign consent for youth participation, and proficiency in English (youth, caregiver) or Spanish (caregiver). Eligible participants were identified by clinic providers who referred interested caregivers and youth to the study team. Participants were interviewed at enrollment and follow-up (FU) time points approximately every 12–18-months by trained research staff. Interviews consisted of psychosocial measures, including a full psychiatric interview (see below). For this analysis, data were obtained from the seventh follow-up interview (FU7), when the participants were between the ages of 20–29, and the GAD-7 was added to the battery. The study was approved by the Institutional Review Board of Columbia University–New York State Psychiatric Institute. All young adults who participated in the study provided written informed consent at FU7 and were reimbursed for their time and travel.

### 2.1. Measures

#### 2.1.1. Sociodemographic information

Demographic factors encompassing age, assigned sex at birth (male or female), HIV status at birth (YAPHIV or YAPHEU), race (African American/Black or other), and ethnicity (Latinx or other), were collected at initial enrollment.

#### 2.1.2. Generalized anxiety disorder measures

The Generalized Anxiety module for the Young Adult version of the Diagnostic Interview Schedule for Children, version IV (DISC-IV-YA) [46] was used to determine GAD diagnoses. The DISC is a widely used, fully structured diagnostic interview that covers the most common disorders of youth and young adults as defined in the American Psychiatric Association’s Diagnostic and Statistical Manual of Mental Disorders (DSM) system, and it has been utilized and validated across a wide range of populations including Black and Latinx groups [46–48]. DISC questions are read to respondents exactly as written by trained research assistants with most responses limited to yes and no, leaving little room for interviewer interpretation. The DISC-IV scoring algorithm determines a positive diagnosis for overall GAD, which is the focus of this analysis.

At FU7 participants were administered the GAD-7 [28] in addition to the DISC. The GAD-7 consists of seven items that assess the severity of anxiety symptoms over the past two weeks, in accordance with DSM-IV criteria for a diagnosis of GAD (e.g., “Feeling nervous, anxious, or on edge”). Participants rate each item on a 4-point Likert scale ranging from 0 (not at all) to 3 (nearly every day), with higher scores indicating greater severity of anxiety symptoms. The total score is calculated, with higher scores indicating higher levels of anxiety. The first two items of the GAD-7 comprise the GAD-2.

### 2.2. Analysis

Sensitivity and specificity of the GAD-7 and the GAD-2 at detecting GAD, using the DISC-IV diagnosis, were first calculated using the recommended cut-points (GAD-7 score ≥ 10; GAD-2 score ≥ 3). Receiver Operating Characteristic (ROC) curves were then constructed using the GAD-7 and GAD-2 as the diagnostic tests of interest for the overall GAD DISC-IV diagnosis. ROC analysis involved calculating AUC (Area Under the Curve) as a measure of the accuracy of each test, while Youden’s Index (J statistic; Sensitivity + Specificity − 1) was used to determine the optimal cut-points. Sensitivity and specificity were also examined for both GAD-7 and GAD-2 for the optimal cut-points. All statistical tests were two-sided and used a p < 0.05 to determine statistical significance. Analyses were completed for the total CASAH population, as well as for the YAPHIV and YAPHEU populations separately. All analyses were conducted using SAS version 9.4.

To further understand differences between the YAPHIV and YAPHEU populations, differential rates of GAD-7 item endorsement by HIV status were assessed using Chi-square and Fisher’s exact tests.

## 3. Results

### 3.1. Demographics

This analysis focused on 204 CASAH participants from FU7, who had DISC-IV information and had a completed GAD-7 and GAD-2 (3 participants were excluded due to missing data), with 62% participants (n = 127) YAPHIV and 38% (n = 77) YAPHEU. Participants were predominantly Black and/or Latinx (45% Black, 38% Latinx, 12% Black Latinx 5% Other), reflecting the full CASAH cohort and the HIV epidemic in women and children in New York City at the time of enrollment [49]. There were no significant differences between the YAPHIV and YAPHEU populations regarding race/ethnicity (p = 0.659). Participants were an average age of 24.1 years (SD = 2.6), and 54% were female. The mean age of YAPHIV was slightly (but statistically significantly) older than YAPHEU (24.4 (2.6) vs. 23.5 (2.6), p = 0.013), as has been found throughout all CASAH FUs after enrollment. There were no statistically significant HIV group differences by sex assigned at birth (p = 0.976). Twenty-five participants in the full sample met diagnostic criteria for GAD (12%), as determined by the DISC, with no significant differences between the YAPHIV and YAPHEU groups (13 [10%] vs. 12 [16%], p = 0.259).

### 3.2. Overall sample

The AUC values using the recommended cut-point for the GAD-7 and GAD-2 were both acceptable (>.80), however they were not optimal. Using the recommended cut-point for GAD-7 (> 10 points), 13 subjects screened positively for overall GAD, whereas 12 subjects screened positively using the recommended cut-point for GAD-2 (>3 points). In this sample, the optimal cut-points determined by our ROC analysis for GAD-7 and GAD-2 were > 6 and > 2 points, respectively). Using these optimal cut-points, 19 participants using the GAD-7 and 20 participants using the GAD-2 screened positive for GAD. The recommended cut-point of 10 points or greater for GAD-7 had a sensitivity of 0.52 (95% CI: 0.31, 0.72) and a specificity of 0.91 (95% CI: 0.86, 0.95) (Table 1); applying the optimal cut-point (>6 points) improved the sensitivity to 0.76 (95% CI: 0.55, 0.91) but sacrificed some specificity (0.77 (95% CI: 0.70, 0.83)). For GAD-2 (>3 points), the sensitivity was 0.48 (95% CI: 0.28, 0.69) and the specificity was 0.90 (95% CI: 0.85, 0.94). Again, applying the optimal cut-point (>2 points), improved the sensitivity (0.80 (95% CI: 0.59, 0.93)), while decreasing the specificity (0.78 (95% CI: 0.71, 0.84)).

### 3.3. Stratified sample

Repeating the ROC analysis by HIV status shows similar accuracy of both the GAD-7 and GAD-2 questionnaires among YAPHIV (GAD-7: AUC=0.86 (95% CI: 0.77, 0.96); GAD-2 0.79 (95% CI: 0.64, 0.93)) and YAPHEU (GAD-7: AUC=0.80 (95% CI: 0.65, 0.95); GAD-2 0.83 (95% CI: 0.70, 0.96)). As shown in Table 2 among YAPHIV, using the optimal cut-points on the GAD-7 and GAD-2 (of 6 and 2, respectively) improves sensitivity uniformly for both instruments (from 0.46 (95% CI: 0.19, 0.75) to 0.77 (95% CI: 0.46, 0.95)). This improvement in sensitivity also was found for YAPHEU using the same optimal cut-points (GAD-7: 0.58 (95% CI: 0.28, 0.85) to 0.75 (95% CI: 0.43, 0.95)); GAD-2: 0.50 (95% CI: 0.21, 0.79) to 0.83 (95% CI: 0.52, 0.98)) (Table 3).

When stratifying by ethnicity, ROC analyses revealed that the optimal cut-point for GAD-7 was > 6 for the Latinx group, but > 7 in the non-Latinx group. There were no differences in the optimal cut-point between ethnicity groups for the GAD-2 score. Lastly, there were no differences in the optimal cut-point between racial groups (Black vs. Non-Black) for either the GAD-7 or GAD-2 scores.

### 3.4. Item response differences by HIV-status

Chi-square and Fisher’s exact tests were used to examine whether there were any differences in endorsement of any of the GAD-7 items by HIV-status. Only one statistically significant difference in GAD-7 item endorsement between YAPHIV and YAPHEU was found (p = 0.004): item 7 “Feeling afraid, as if something awful might happen.” YAPHIV endorsed the “Several Days” response (score of 1 on Likert scale) significantly more (29 (22.8%) vs. 4 (5.2%)) than YAPHEU, (p < 0.001), and the “Not at all” response significantly less than the YAPHEU (88 (69.3%) vs 64 (83.1%), p = 0.028).

### 3.5. Discussion

Anxiety disorder prevalence is notably elevated in young people affected by HIV. Brief screeners such as the GAD-7 and GAD-2 have shown great utility in identifying those at-risk in busy or resource-limited clinical settings. However, these screeners have not been extensively validated for use among diverse populations, including Black and Latinx young adults in general and particularly in those perinatally-affected by HIV. This study examined the accuracy of GAD-7 and GAD-2 cut-points of > 10 and > 3, respectively, to identify GAD among a sample of primarily Black and Latinx YAPHIV and YAPHEU. When compared with a widely used psychiatric diagnostic interview, our results indicate that the commonly recommended cut-points may be too high to identify probable GAD in minoritized populations affected by HIV. Lower cut-points might be important for identifying those most at risk for anxiety. These findings are similar to previous work done examining cut-points when screening for depression in young minoritized populations in the United States affected by HIV [43].

We found the GAD-7 had only a sensitivity of.52, meaning nearly half of all participants with a GAD diagnosis did not score above the recommended clinical cut-point of 10. Sensitivity was similarly low for the GAD-2. Lowering the cut-points of the GAD-7 and GAD-2 to > 6 and > 2, respectively, increased their sensitivity to detect GAD; however, this in turn decreased the specificity of both screeners from.91 to.77 on the GAD-7 and from.89 to.77 on the GAD-2. If the aim of an anxiety screener is to maximize identification of those at-risk for GAD, then maximizing sensitivity at the expense of specificity may be an acceptable tradeoff, because early intervention for GAD has been found to be vital in reducing negative mental health outcomes [50,51]. Primary care settings are ideal for routine screening for mental health disorders with at-risk groups [27,52]. Notably, our results found comparable sensitivity and specificity between the GAD-7 and GAD-2 in our sample, suggesting the even briefer screener may be a valuable and effective tool in busy or low-resource clinic settings. Early identification of youth/young adults affected by HIV who are at risk for GAD may promote positive mental and behavioral health outcomes and prevent worse outcomes through timely linkage to care. However, using the GAD-7 and GAD-2 in these populations may require adjusting the recommended cut-points to optimize their sensitivity.

Overall, our analyses showed that there were no significant differences in performance of the GAD-7 and GAD-2 for the YAPHIV and YAPHEU subgroups. Both groups come from similar sociodemographic backgrounds (e.g. resource-limited communities affected by violence, substance use and neighborhood stressors) [22,53,54]. Thus, any contextual, racial/ethnic, or cultural biases of the GAD-7 and GAD-2 are likely to similarly impact both groups. When looking at differences in GAD-7 item endorsement by HIV status, only one item was significantly different: YAPHIV were more likely to endorse “feeling afraid, as if something awful might happen” on “several days.” Being born with HIV, a highly stigmatized chronic illness, may cause YAPHIV to feel that they are at increased risk of something bad happening to them. Future studies should include a Black/Latinx non-HIV-affected subgroup to better understand the effect of HIV-infection or exposure on GAD-7 and GAD-2 performance and item endorsement.

### 3.6. Limitations

This study’s findings must be considered in the context of its limitations. Although our study sample is reflective of the recruitment clinics and the US pediatric HIV epidemic at the time, the overall number of participants in our study sample is relatively small given the prevalence of perinatally-acquired HIV in the US. Moreover, although we found that anxiety disorders are more widespread in our participants than mood disorders, the number of participants meeting criteria for a GAD diagnosis on the DISC-IV is still relatively small. Thus, our ability to detect the predictive validity of the cut-points in identifying GAD may have been limited, particularly when examining ethnic, racial, or gender subgroup differences. Given the epidemiology of HIV in women and children in the U.S. (i.e., predominantly in Black and Latinx populations), the study participants were predominantly Black and Latinx; therefore, it is difficult to draw direct conclusions regarding the influence of race and ethnicity or perinatal HIV-exposure on optimized GAD-7 cut-points without a Black/Latinx non-HIV affected comparison group. Previous literature on GAD in Black/Latinx people living in New York City have found a comparable prevalence of GAD (12%) [55], however future studies are comparing GAD-7 performance between HIV and non-HIV affected Black/Latinx participants are needed. Also, given the elevated prevalence of adolescent HIV (e.g., perinatal or behavioral transmission) in Sub-Saharan Africa [56], further GAD assessment in these regions would be helpful. Lastly, although both the GAD-7 and GAD modules on the DISC-IV were developed using DSM-IV and not DSM-V criteria for GAD, there were no changes in GAD criteria between DSM-IV and DSM-V [4].

### 3.7. Conclusions

There have been many calls for integrating mental health screening and treatment into HIV care for all populations, necessitating brief, validated screening tools [3]. Screeners such as the GAD-7 and GAD-2 take only one to two minutes to administer- a significantly shorter administration time than other anxiety diagnostic tools that may take upwards of 20 min [57]. Therefore, it is an efficient way to identify those at risk for impairing anxiety disorders. This shortened time frame can be critically important to increasing the feasibility of mental health screening, particularly in resource-constrained settings, including busy HIV care clinics. Our data support the use of GAD-7 and GAD-2 but suggest utilizing lower cut-points for GAD screening in young adults perinatally affected by HIV. This finding is similar to a prior analysis of the validity of using the standard cut-scores on the PHQ-9 as a depression screener for the same participant population [43]. These findings, along with those cited in Mufson et al. (2022), suggest that prioritizing sensitivity in mental health screening instruments may maximize their clinical utility for identifying at-risk patients and facilitating earlier linkage to care [43]. Future studies with larger sample sizes, more diverse ethnic/racial comparison groups, and a non-HIV affected comparison group would be helpful in identifying the impact of specific demographic characteristics on GAD-7 performance. This would enable more precise optimization of the GAD-7 and GAD-2 cut-points for use among young adults perinatally affected by HIV, thereby ensuring effective anxiety screening in this population and prevention of increasing psychiatric disorders and impairment.

## Figures and Tables

**Table 1:** Comparing sensitivity and specificity of recommended and optimal cut-points for GAD-7 & GAD-2 in YAPHIV and YAPHEU (n = 204).

Recommended Cut-point					Optimal Cut-point			
	GAD Diagnosis on DISC-IV (N)	Score	Sensitivity (95% CI)	Specificity (95% CI)	Score	Sensitivity (95% CI)	Specificity (95% CI)	AUC (95% CI)
**GAD-7**	25	10	0.52 (0.31, 0.72)	0.91 (0.86, 0.95)	6	0.76 (0.55, 0.91)	0.77 (0.70, 0.83)	0.83 (0.74, 0.92)
**GAD-2**	25	3	0.48 (0.28, 0.69)	0.90 (0.85, 0.94)	2	0.80 (0.59, 0.93)	0.78 (0.71, 0.84)	0.82 (0.72, 0.91)

**Table 2:** Comparing sensitivity and specificity of recommended and optimal cut-points for GAD-7 & GAD-2 in YAPHIV (n = 127).

*Recommended Cut-Point*					*Optimal Cut-Point*			
	GAD Diagnosis on DISC-IV (N)	Score	Sensitivity (95% CI)	Specificity (95% CI)	Score	Sensitivity (95% CI)	Specificity (95% CI)	AUC (95% CI)
GAD-7	13	10	0.46 (0.19, 0.75)	0.92 (0.86, 0.96)	6	0.77 (0.46, 0.95)	0.76 (0.67, 0.84)	0.80 (0.65, 0.95)
GAD-2	13	3	0.46 (0.19, 0.75)	0.92 (0.86, 0.96)	2	0.77 (0.46, 0.95)	0.79 (0.70, 0.86)	0.83 (0.70, 0.96)

**Table 3:** Comparing sensitivity and specificity of recommended and optimal cut-points for GAD-7 & GAD-2 in YAPHEU (n = 77).

*Recommended Cut-Point.*					*Optimal Cut-Point*			
	GAD Diagnosis on DISC-IV (N)	Score	Sensitivity (95% CI)	Specificity (95% CI)	Score	Sensitivity (95% CI)	Specificity (95% CI)	AUC (95% CI)
GAD-7	12	10	0.58 (0.28, 0.85)	0.89 (0.79, 0.96)	6	0.75 (0.43, 0.95)	0.83 (0.72, 0.91)	0.86 (0.77, 0.96)
GAD-2	12	3	0.50 (0.21, 0.79)	0.86 (0.75, 0.93)	2	0.83 (0.52, 0.98)	0.75 (0.63, 0.85)	0.79 (0.64, 0.93)

## Abbreviations:

CASAH: Child and Adolescent Self Awareness and Health Study
YAPHEU: Young adults perinatally HIV exposed but not infected
YAPHIV: Young adults with perinatally-acquired HIV

## References

[1] Mannes ZL, Hearn LE, Zhou Z, Janelle JW, Cook RL, Ennis N. The association between symptoms of generalized anxiety disorder and appointment adherence, overnight hospitalization, and emergency department/urgent care visits among adults living with HIV enrolled in care. J Behav Med 2019 Apr 1;42(2):330–41. 30387009 10.1007/s10865-018-9988-6 PMC6447438

[2] Mannes ZL, Dunne EM, Ferguson EG, Cook RL, Ennis N. Symptoms of generalized anxiety disorder as a risk factor for substance use among adults living with HIV. AIDS Care 2021 May;33(5):623–32. 32835502 10.1080/09540121.2020.1808163 PMC7902737

[3] Remien RH, Stirratt MJ, Nguyen N, Robbins RN, Pala AN, Mellins CA. Mental health and HIV/AIDS: the need for an integrated response. AIDS 2019 Jul 15;33(9):1411–20. 30950883 10.1097/QAD.0000000000002227 PMC6635049

[4] American Psychiatric Association. Anxiety disorders: DSM-5^®^ Selections. American Psychiatric Pub; 2015: p. 130.

[5] Chaudhury S, Bakhla AK, Saini R. Prevalence, impact, and management of depression and anxiety in patients with HIV: a review. NBHIV 2016 May 5;7:15–30.

[6] Kessler RC, Berglund P, Dernier O, Jin R, Merikangas KR, Walters EE. Lifetime prevalence and age-of-onset distributions of DSM-IV disorders in the National Comorbidity Survey Replication. Arch Gen Psychiatry 2005 Jun;62(6):593–602. 15939837 10.1001/archpsyc.62.6.593

[7] Henning ER, Turk CL, Mennin DS, Fresco DM, Heimberg RG. Impairment and quality of life in individuals with generalized anxiety disorder. Depress Anxiety 2007;24(5):342–9. 17091478 10.1002/da.20249

[8] Wittchen HU, Carter RM, Pfister H, Montgomery SA, Kessler RC. Disabilities and quality of life in pure and comorbid generalized anxiety disorder and major depression in a national survey. Int Clin Psychopharmacol 2000 Nov;15(6):319–28. 11110007 10.1097/00004850-200015060-00002

[9] Rane MS, Hong T, Govere S, Thulare H, Moosa MY, Celum C, Depression and anxiety as risk factors for delayed care-seeking behavior in human immunodeficiency virus-infected individuals in South Africa. Clin Infect Dis 2018 Oct 15;67(9):1411–8. 29659757 10.1093/cid/ciy309 PMC6186861

[10] Ruiz MA, Zamorano E, García-Campayo J, Pardo A, Freire O, Rejas J. Validity of the GAD-7 scale as an outcome measure of disability in patients with generalized anxiety disorders in primary care. J Affect Disord 2011 Feb;128(3):277–86. 20692043 10.1016/j.jad.2010.07.010

[11] Psaros C, O’Cleirigh C, Bullis JR, Markowitz SM, Safren SA. The influence of psychological variables on health related quality of life among HIV positive individuals with a history of intravenous drug use. J Psychoact Drugs 2013;45(4):304–12. 10.1080/02791072.2013.825030 PMC3877931 24377169

[12] Stanley S, Sethuramalingam V, Sathia S. Quality of life correlates in HIV-positive people in a city in south India. J HIV/AIDS Soc Serv 2014 Oct 2;13(4):337–52.

[13] Zimpel RR, Fleck MP. Depression as a major impact on the quality of life of HIV-positive Brazilians. Psychol Health Med 2014;19(1):47–58. 23458241 10.1080/13548506.2013.772302

[14] Beer L, Tie Y, Padilla M, Shouse RL. Generalized anxiety disorder symptoms among persons with diagnosed HIV in the United States. AIDS 2019 Sep 1;33(11):1781. 31211718 10.1097/QAD.0000000000002286 PMC6663599

[15] Murphy DA, Durako SJ, Moscicki AB, Vermund SH, Ma Y, Schwarz DF, No change in health risk behaviors over time among HIV infected adolescents in care: role of psychological distress. J Adolesc Health 2001 Sep 1;29(3):57–63. 11530304 10.1016/s1054-139x(01)00287-7

[16] Nguyen N, Choi CJ, Robbins R, Korich R, RAYMOND J, Dolezal C, Psychiatric trajectories across adolescence in perinatally HIV-exposed youth: the role of HIV infection and associations with viral load. AIDS 2020 Jul 1;34(8):1205–15. 32287067 10.1097/QAD.0000000000002529 PMC7554128

[17] Vreeman RC, McCoy BM, Lee S. Mental health challenges among adolescents living with HIV. J Int AIDS Soc 2017;20(S3):21497. 28530045 10.7448/IAS.20.4.21497 PMC5577712

[18] Centers for Disease Control and Prevention. Pediatric HIV surveillance 2018 [Internet]; 2018. Available from: 〈https://www.cdc.gov/hiv/pdf/library/slidesets/cdc-hiv-surveillance-pediatric-2018.pdf〉.

[19] Centers for Disease Control and Prevention. Centers for Disease Control and Prevention [cited 2023 Oct 22]. Pregnant women, infants, and children/gender/HIV by group/HIV/AIDS/CDC; 2019. Available from: 〈https://www.cdc.gov/hiv/group/pregnant-people/index.html〉.

[20] Lijster JM de, Dierckx B, Utens EMWJ, Verhulst FC, Zieldorff C, Dieleman GC, The age of onset of anxiety disorders: a meta-analysis. Can J Psychiatry 2017 Apr 1;62(4):237–46. 27310233 10.1177/0706743716640757 PMC5407545

[21] Centers for Disease Control and Prevention. Centers for Disease Control and Prevention [cited 2021 Aug 24]. Cases, data, and surveillance; 2020. Available from: 〈https://www.cdc.gov/coronavirus/2019-ncov/covid-data/investigations-discovery/hospitalization-death-by-race-ethnicity.html〉.

[22] Kang E, Mellins CA, Dolezal C, Elkington KS, Abrams EJ. Disadvantaged neighborhood influences on depression and anxiety in youth with perinatally acquired human immunodeficiency virus: how life stressors matter. J Community Psychol 2011;39(8):956–71. 23472046 10.1002/jcop.20483 PMC3587679

[23] Kang E, Mellins CA, Kim W, Dolezal C, Kindler C, Leu CS, Navigating stigma trajectory and mental health among young adults living with perinatal HIV in New York City. AIDS Behav 2021 Nov;25(11):3712–20. 33523346 10.1007/s10461-021-03166-3 PMC8638371

[24] Mellins CA, Malee KM. Understanding the mental health of youth living with perinatal HIV infection: lessons learned and current challenges. J Int AIDS Soc 2013 Jun 18;16:18593. 23782478 10.7448/IAS.16.1.18593 PMC3687078

[25] Smith R, Huo Y, Tassiopoulos K, Rutstein R, Kapetanovic S, Mellins C, Mental health diagnoses, symptoms, and service utilization in US youth with perinatal HIV infection or HIV exposure. AIDS Patient Care STDs 2019 Jan;33(1):1–13. 30601062 10.1089/apc.2018.0096 PMC6338457

[26] Combs H, Markman J. Anxiety disorders in primary care. Med Clin 2014;98(5):1007–23. 10.1016/j.mcna.2014.06.003 25134870

[27] Rosner S. Increasing the recognition of generalized anxiety disorder in primary care. Fam Med Clerkship Stud Proj 2015 Jan 1. Available from: 〈https://scholarworks.uvm.edu/fmclerk/48〉.

[28] Spitzer RL, Kroenke K, Williams JBW, Löwe B. A brief measure for assessing generalized anxiety disorder: the GAD-7. Arch Intern Med 2006 May 22;166(10):1092–7. 16717171 10.1001/archinte.166.10.1092

[29] Bauermeister JA, Elkington K, Brackis-Cott E, Dolezal C, Mellins C. Sexual behavior and perceived peer norms: comparing perinatally infected and affected youth. J Youth Adolesc 2009 Sep;38(8):1110–22. 19636775 10.1007/s10964-008-9315-6 PMC2769517

[30] Abrams EJ, Mellins CA, Bucek A, Dolezal C, Raymond J, Wiznia A, Behavioral health and adult milestones in young adults with perinatal HIV infection or exposure. Pediatrics 2018 Sep 1;142(3):e20180938. 30097528 10.1542/peds.2018-0938 PMC6317560

[31] Mellins CA, Tassiopoulos K, Malee K, Moscicki AB, Patton D, Smith R, Behavioral health risks in perinatally HIV-exposed youth: co-occurrence of sexual and drug use behavior, mental health problems, and nonadherence to antiretroviral treatment. AIDS Patient Care STDs 2011 Jul;25(7):413–22. 21992620 10.1089/apc.2011.0025 PMC3125549

[32] Kroenke K, Spitzer RL, Williams JBW. The PHQ-9. J Gen Intern Med 2001;16(9):606–13. 11556941 10.1046/j.1525-1497.2001.016009606.x PMC1495268

[33] Kroenke K, Spitzer RL, Williams JBW, Monahan PO, Löwe B. Anxiety disorders in primary care: prevalence, impairment, comorbidity, and detection. Ann Intern Med 2007 Mar 6;146(5):317–25. 17339617 10.7326/0003-4819-146-5-200703060-00004

[34] Löwe B, Decker O, Müller S, Brähler E, Schellberg D, Herzog W, Validation and standardization of the Generalized Anxiety Disorder Screener (GAD-7) in the general population. Med Care 2008;46(3):266–74. 18388841 10.1097/MLR.0b013e318160d093

[35] First MB, Gibbon M, Spitzer RL, Williams JB, Benjamin LS. Structured clinical interview for DSM-IV^®^ axis ii personality disorders SCID-II. American Psychiatric Pub; 1997.

[36] Romano I, Ferro MA, Patte KA, Leatherdale ST. Measurement invariance of the GAD-7 and CESD-R-10 among adolescents in Canada. J Pediatr Psychol 2022 Jun 1;47(5):585–94. 35552429 10.1093/jpepsy/jsab119 PMC9113328

[37] Hinz A, Klein AM, Brähler E, Glaesmer H, Luck T, Riedel-Heller SG, Psychometric evaluation of the Generalized Anxiety Disorder Screener GAD-7, based on a large German general population sample. J Affect Disord 2017 Mar 1;210:338–44. 28088111 10.1016/j.jad.2016.12.012

[38] Byrd-Bredbenner C, Eck K, Quick V. Psychometric properties of the generalized anxiety disorder-7 and generalized anxiety disorder-mini in United States university students. Front Psychol 2020 Sep 24,11:550533. 33071867 10.3389/fpsyg.2020.550533 PMC7541941

[39] Parkerson HA, Thibodeau MA, Brandt CP, Zvolensky MJ, Asmundson GJG. Cultural-based biases of the GAD-7. J Anxiety Disord 2015 Apr 1;31:38–42. 25725310 10.1016/j.janxdis.2015.01.005

[40] Gutiérrez-Velilla E, Barrientos-Casarrubias V, Cruz-Maycott R, Perrusquia-Ortiz LE, Alvarado-de la Barrera C, Ávila-Ríos S, Assessment of anxiety in Mexican persons living with HIV using a culturally-adapted version of the GAD-7 test. J Health Psychol 2022 Nov;27(13):2875–86. 35042393 10.1177/13591053211072687

[41] Schulte MT, Marelich WD, Payne DL, Tarantino N, Armistead LP, Murphy DA. Validation of a brief measure of HIV health-related anxiety among women living with HIV. Res Nurs Health 2018 Jun;(4). 10.1002/nur.21876. PMC6557685 29862527

[42] Shacham E, Morgan JC, Önen NF, Taniguchi T, Overton ET. Screening anxiety in the HIV clinic. AIDS Behav 2012 Nov 1;16(8):2407–13. 22718040 10.1007/s10461-012-0238-6 PMC3623696

[43] Mufson L, Morrison C, Shea E, Kluisza L, Robbins R, Chen Y, Screening for depression with the PHQ-9 in young adults affected by HIV. J Affect Disord 2022 Jan 15;297:276–82. 34695500 10.1016/j.jad.2021.10.037 PMC9762407

[44] Mellins CA, Brackis-Cott E, Leu CS, Elkington KS, Dolezal C, Wiznia A, Rates and types of psychiatric disorders in perinatally human immunodeficiency virus-infected youth and seroreverters. J Child Psychol Psychiatry 2009;50(9):1131–8. 19298479 10.1111/j.1469-7610.2009.02069.x PMC2775808

[45] Mellins CA, Elkington KS, Leu CS, Santamaria EK, Dolezal C, Wiznia A, Prevalence and change in psychiatric disorders among perinatally HIV-infected and HIV-exposed youth. AIDS Care 2012 Aug;24(8):953–62. 22519762 10.1080/09540121.2012.668174 PMC3416047

[46] Shaffer D, Fisher P, Lucas CP, Dulcan MK, Schwab-Stone ME. NIMH Diagnostic Interview Schedule for Children Version IV (NIMH DISC-IV): description, differences from previous versions, and reliability of some common diagnoses. J Am Acad Child Adolesc Psychiatry 2000 Jan;39(1):28–38. 10638065 10.1097/00004583-200001000-00014

[47] Kim IJ, Ge X, Brody GH, Conger RD, Gibbons FX, Simons RL. Parenting behaviors and the occurrence and co-occurrence of depressive symptoms and conduct problems among African American children. J Fam Psychol 2003;17(4):571–83. 14640806 10.1037/0893-3200.17.4.571

[48] Yeh M, McCabe K, Hough RL, Dupuis D, Hazen A. Racial/ethnic differences in parental endorsement of barriers to mental health services for youth. Ment Health Serv Res 2003 Jun 1;5(2):65–77. 12801070 10.1023/a:1023286210205

[49] New York City Department of Health and Mental Hygiene. Pediatric and adolescent HIV/AIDS Surveillance update. Department of Health and Mental Hygiene; New York City; 2007.

[50] Donovan CL, Spence SH. Prevention of childhood anxiety disorders. Clin Psychol Rev 2000 Jun;20(4):509–31. 10832552 10.1016/s0272-7358(99)00040-9

[51] Lynn C, Bradley-Klug K, Chenneville TA, Walsh AStJ, Dedrick R, Rodriguez C. Mental health screening in integrated care settings: identifying rates of depression, anxiety, and posttraumatic stress among youth with HIV. J HIV/AIDS Soc Serv 2018 Jul 3;17(3):239–45.

[52] Bandelow B, Michaelis S. Epidemiology of anxiety disorders in the 21st century. Dialog Clin Neurosci 2015 Sep 30;17(3):327–35. 10.31887/DCNS.2015.17.3/bbandelow PMC4610617 26487813

[53] Boyd-Franklin NE, Steiner GL, Boland MG. Children, families, and HIV/AIDS: psychosocial and therapeutic issues. The Guilford Press; 1995.

[54] Elkington KS, Bauermeister JA, Santamaria EK, Dolezal C, Mellins CA. Substance use and the development of sexual risk behaviors in youth perinatally exposed to HIV. J Pediatr Psychol 2015 May 1;40(4):442–54. 25476800 10.1093/jpepsy/jsu103 PMC4405197

[55] Lee JY, Brook JS, Finch SJ, De La Rosa M, Brook DW. Joint trajectories of cigarette smoking and depressive symptoms from the mid-20s to the mid-30s predicting generalized anxiety disorder. J Addict Dis 2017 Jul-Sep;36(3):158–66. 10.1080/10550887.2017.1303958. Epub 2017 Mar 10. 28281938 PMC5503101

[56] UNICEF. UNICEF data. [cited 2023 Sep 28]. Adolescent HIV prevention: turning the tide against AIDS will require more concentrated focus on adolescents and young people; 2021. Available from: 〈https://data.unicef.org/topic/hivaids/global-regional-trends/〉.

[57] Mossman Sarah A, Luft Marissa J, Schroeder Heidi K, Varney Sara T, Fleck David E, The Generalized Anxiety Disorder 7-item (GAD-7) scale in adolescents with generalized anxiety disorder: signal detection and validation. Ann Clin Psychiatry 2017 Nov;29(4):227–234A. 29069107 PMC5765270

